# Exploring the Dimensions of Perfectionism in Adolescence: A Multi-Method Study on Mental Health and CBT-Based Psychoeducation

**DOI:** 10.3390/brainsci15010091

**Published:** 2025-01-19

**Authors:** Magdalena Chęć, Krystian Konieczny, Sylwia Michałowska, Karolina Rachubińska

**Affiliations:** Department of Clinical Psychology and Psychoprophylaxis, Institute of Psychology, University of Szczecin, 71-017 Szczecin, Poland; magdalena.chec@usz.edu.pl (M.C.); krystian.konieczny@usz.edu.pl (K.K.); sylwia.michalowska@usz.edu.pl (S.M.)

**Keywords:** perfectionism, adolescence, mental health, psychoeducation, school environment

## Abstract

Background: Perfectionism in adolescents can have both adaptive and maladaptive forms, with implications for mental health and school performance. This study aimed to investigate the relationships among perfectionism, mental health factors, and emotional regulation in adolescents and to evaluate the efficacy of psychoeducational interventions. Methods: Two studies were conducted: (1) A cross-sectional study (*n* = 261) examined the correlations among perfectionism, mental health factors, and emotional regulation. (2) An experimental study (*n* = 115) evaluated the effects of psychoeducation on perfectionism and healthy habits compared with a control group. The measures included questionnaires on perfectionism, depression, anxiety, stress, and emotional regulation. Results: Study 1 found that maladaptive perfectionism was positively correlated with depression (r = 0.52, *p* < 0.001), anxiety (r = 0.48, *p* < 0.001), stress (r = 0.45, *p* < 0.001), and difficulties in emotional regulation (r = 0.39, *p* < 0.001). Adaptive perfectionism was negatively correlated with deficits in emotional understanding (r = −0.31, *p* < 0.05). Study 2 showed that psychoeducational interventions reduced maladaptive perfectionism (mean difference = −5.7, *p* < 0.05, Cohen’s d = 0.62) and depression levels (mean difference = −3.2, *p* < 0.05, Cohen’s d = 0.38) but increased stress in the experimental groups. No significant changes were observed in adaptive perfectionism or the anxiety level. Conclusions: These findings highlight the complex relationships among perfectionism, mental health, and emotional regulation in adolescents. Targeted interventions can reduce maladaptive perfectionism and its associated negative effects. Further research is needed on the long-term outcomes and refinement of interventional strategies.

## 1. Introduction

### 1.1. Theoretical Foundations of This Study

Perfectionism is an inner need to achieve perfection or to give that impression in one’s own and other people’s eyes [1], and it has been demonstrated to be an exceedingly complex phenomenon. More than four decades ago, Hamachek (1978) categorised perfectionists into two distinct groups: healthy and unhealthy. The healthy dimension of perfectionism is characterised by flexibility of action, the ability to modify established goals, acceptance of mistakes, deriving pleasure from undertaken activities, and anticipation before commencing a task. Conversely, the unhealthy type of perfectionism is characterised by excessively high and rigid standards and a persistent sense of inadequacy, and, consequently, an inability to experience satisfaction, setting unattainable goals, and experiencing fear of failure as a typical emotion prior to taking action. In 1990, Frost delineated six components of perfectionism based on Hamachek’s theory. These include excessive concern over mistakes, high personal standards, doubts about actions, the need for organisation, high parental expectations, and excessive parental criticism [2]. The outcome of these concepts was the distinction between the two subtypes of perfectionism, which correlated with the experience of desirable and undesirable emotions [3]. A differentiation has been established between adaptive perfectionism, originally described by Frost as perfectionistic striving, and maladaptive perfectionism, initially termed as perfectionistic fear [4]. The development of a particular subtype of perfectionism remains strongly associated with the type of goals an individual establishes for themselves and their approach to pursuing them [5].

### 1.2. Connections Among Perfectionism, the School Environment, and Mental Health

Individuals exhibiting adaptive perfectionism demonstrate high personal standards and a need for organisation, which facilitate the pursuit of established goals. However, if individuals focus on high expectations and frequent criticism and place excessive importance on their mistakes, they will manifest maladaptive perfectionism [5]. The school environment has been identified as significant in this context [6] for two primary reasons: First, it serves as a setting in which the manifestation of perfectionism can be observed. Second, research indicates that the school environment plays a crucial role in the development of perfectionist traits [7]. Perfectionism affects both an individual’s functioning within the school environment and their mental health. Students with adaptive perfectionism, characterised by high standards and a need for good organisation, tend to cope better with academic challenges and achieve better educational outcomes, which contribute to their well-being [8,9]. In contrast, maladaptive perfectionism, associated with fear of failure and a focus on mistakes, leads to procrastination, lower motivation, and higher stress levels. As a result, these students are more likely to experience school anxiety and emotional difficulties, such as rumination and depression [10,11].

The school environment plays a key role in shaping perfectionism. On the one hand, as a place of intense social interactions, it can reinforce the adaptive aspects of perfectionism, such as the motivation to achieve mastery. On the other hand, excessive teacher demands, peer pressure, and competition may contribute to the development of maladaptive perfectionism, increasing the risk of mental health problems [6,7].

In terms of mental health, research shows that maladaptive perfectionism is a significant risk factor for emotional disorders, such as anxiety and depression [12,13,14]. These mechanisms include a lack of effective emotional regulation strategies, leading to worry, negative rumination, and lowered mood [15,16]. For students with adaptive perfectionism, positive school experiences may support the development of emotional competencies and better psychological functioning.

### 1.3. Scientific Research Conducted on the Analysed Variables

Previous research has demonstrated that maladaptive perfectionism among adolescents is negatively correlated with their school performance [10]. Studies have shown that students characterised by maladaptive perfectionism engage in learning, motivated by a desire to avoid failure, which subsequently leads to procrastination. Conversely, students exhibiting adaptive perfectionism are motivated by a desire to achieve mastery [8]. Findings from the analyses suggest that perfectionistic aspirations have the potential to support educational success, whereas perfectionistic fears may impede it [9]. Concurrently, school achievement and self-efficacy predict an increase in perfectionistic striving [17].

The existence of gender differences in perfectionism has been proven among school children. Studies have indicated that girls in elementary school are more prone to perfectionism than their male peers [18]. According to some studies, these differences disappear with age, especially in late adolescence [19]. Conclusions in this regard are inconsistent; however, research studies have shown that girls generally show higher levels of perfectionism than boys [20]. Social norms related to gender roles, which shape expectations for men and women, can, therefore, influence the perception and experience of perfectionism and its impact on mental health. Women may be socialised towards meeting the expectations of others and are more likely to set high standards for their relationships [21]. Parental attitudes, both from fathers and mothers, also play a significant role in shaping perfectionism in women and men. According to the analyses, for women, paternal overprotection was positively associated with socially prescribed perfectionism. In contrast, for men, maternal overprotection was positively linked to socially prescribed perfectionism [22]. Age may also be a significant factor. Melero et al. (2020) found that younger children had higher levels of social perfectionism and lower levels of critical self-oriented perfectionism than adolescents [23]. This is reasonable, given the understanding of perfectionism as a personality trait that develops and evolves over a lifetime. In childhood, perfectionism is often the result of expectations of the environment, and its main form is “self-directed perfectionism”. Young children begin to adopt social norms and expectations from parents and teachers, which can lead to a tendency towards self-criticism and fear of failure. Perfectionism intensifies during adolescence. Adolescents are more exposed to social comparison and peer pressure, which reinforce the need to strive for the ideal. According to Hewitt and Flett’s (1991) theory of self-critical perfectionism, young people tend to idealise school, sports, or social performance and treat any deviation from their own standards as a personal failure [24].

A study comparing perfectionistic striving, perfectionistic fears, and mixed-type perfectionism, conducted on a group of more than 1500 students between the ages of 12 and 18, found that both perfectionistic striving and perfectionistic fears were associated with experiencing school anxiety, but students with perfectionistic fears exhibited a consistent pattern in terms of this anxiety [10]. Similar findings emerged from a study on exam anxiety, which revealed that students with perfectionistic fears experienced this anxiety the most acutely [11]. Contemporary research analyses suggest both general and specific links between maladaptive perfectionism and the symptoms of anxiety disorders among adolescents [12,25,26]. Associations between perfectionism exhibited by adolescents and worry, rumination, and depressive symptoms have also been demonstrated [27,28,29,30]. The risk of mental health disorders among young individuals exhibiting perfectionism may be attributed to a deficiency in effective emotional regulation strategies, with ruminations being predominant [15]. Vincent et al. (2022) indicate that individuals with high perfectionism may be more susceptible to developing maladaptive emotional self-regulation strategies, such as ruminations, because of their propensity to excessively focus on negative emotional experiences [31]. Furthermore, research has demonstrated that a maladaptive form of perfectionism among students is associated with depression [13]. Smith et al. (2021) elucidated that the relationship between perfectionistic worries and depressive symptoms is reciprocal. Perfectionistic fears can predict the severity of depressive symptoms and vice versa [32]. Conversely, the association between perfectionistic striving and depressive symptoms is unidirectional. This relationship can be attributed to the maladaptive coping style of individuals with high levels of perfectionism, which results in an increased propensity to experience negative emotional states, including depression [33]. A longitudinal study conducted in China has revealed a significant direct effect of perfectionism on depressive symptoms. Notably, self-compassion and the imposter phenomenon function as mediators of this relationship [34]. Analyses conducted on a cohort of adolescents demonstrated a correlation between repetitive negative thinking and lowered moods. This relationship is more pronounced in a group of adolescents exhibiting higher levels of perfectionism [16]. Recent scientific analyses confirm the destructive impact of perfectionism on the mental health of adolescents [35,36,37,38].

In response to the potential adverse consequences of maladaptive perfectionism in adolescents, including increased stress [39], anxiety [40], social withdrawal [41], difficulty in regulating emotions [42], and risk of depression [43], researchers have sought effective therapeutic and preventive interventions. Among the most well-documented approaches to the therapy and prevention of perfectionism are methods based on cognitive–behavioural therapy [11,44,45,46]. Cognitive–behavioural theory’s conceptualisation of perfectionism posits that its fundamental components comprise a fear of failure, a persistent pursuit of success, and a sense of self-worth that is heavily contingent upon achievement. This manifests as inflexible standards in the form of beliefs and rules that govern one’s life, such as “one must never make mistakes” [47]. Limited flexibility in perceiving high standards results in cognitive distortions, including dichotomous thinking, overgeneralisation, and selective attention. Additionally, behaviours associated with performance verification, social comparison, and seeking reassurance regarding adequacy are prevalent. According to the cognitive model, these factors collectively perpetuate perfectionism-related issues [48]. Standard therapy in the cognitive–behavioural stream encompasses psychoeducation, which enables individuals to comprehend the mechanisms of perfectionism and facilitates the disruption of the vicious cycle present in cognition and behaviour [49]. Steele et al. (2013) indicated that group cognitive–behavioural therapy can mitigate symptoms of clinical perfectionism, whereas psychoeducation alone is not efficacious in this regard [50]. Subsequent studies, however, have demonstrated that one-session interventions targeting perfectionism yield promising results and warrant further investigation [51]. An experiment conducted by Bento et al. (2017), evaluating the effectiveness of a single session of cognitive–behavioural group psychoeducation on perfectionism, revealed that these interactions can contribute to a reduction in perfectionism severity among adolescents, with effects observed at two and six months post intervention [52]. Numerous studies have corroborated the efficacy of cognitive–behavioural group sessions [53,54], including those with a psychoeducational focus [55,56].

The findings of the cited studies indicate a significant relationship between perfectionism and emotional problems, as well as mental health issues among adolescents, emphasising the need for the implementation of specific actions to support young people in managing these difficulties. Interventions based on cognitive–behavioural therapy, including psychoeducational group sessions that help participants to understand the mechanisms of perfectionism and modify harmful thought patterns, could potentially serve as an effective form of support. Such programmes should focus on skills for coping with fear of failure, eliminating unrealistic standards, and strengthening positive self-esteem, enabling young people to better manage their expectations and emotional responses. Furthermore, interventions that develop emotional regulation skills—such as relaxation techniques, mindfulness, and strategies for coping with rumination—may help to alleviate anxiety and depressive symptoms associated with perfectionism. Based on studies such as those by Bento et al. (2017) and Egan et al. (2022), which demonstrated positive effects of psychoeducational programmes in reducing perfectionism and improving mental health among adolescents, it is recommended to implement such programmes in schools and institutions that support young people [46,52]. The practical application of those research findings could provide an effective tool to counteract the negative consequences of perfectionism, such as stress, anxiety, or depression, and, ultimately, contribute to improving the mental well-being of young people.

### 1.4. Objectives of the Present Study

To investigate the relationships among perfectionism (adaptive and maladaptive), mental health factors (anxiety, stress, and depression), and emotional regulation among adolescents;

To identify predictors of adaptive and maladaptive perfectionism in students;

To evaluate the efficacy of psychoeducation on perfectionism and healthy habits in altering the severity of maladaptive and adaptive perfectionisms, as well as symptoms such as depression, anxiety, and stress;

To compare the effectiveness of psychoeducation on perfectionism versus healthy habits in modifying these factors.

Hypotheses for Research 1 (Cross-sectional study):

Perfectionism is related to mental health factors (e.g., depression, stress, and anxiety) in adolescents;

There is an association between perfectionism and emotional regulation in adolescents;

Mental health factors (depression, stress, and anxiety) are related to emotional regulation in adolescents.

Hypotheses for Research 2 (Experimental study):

Psychoeducation on perfectionism will result in an increase in adaptive perfectionism compared to the healthy-habit and control groups;

Psychoeducation on perfectionism will lead to a decrease in maladaptive perfectionism compared to the healthy-habit and control groups;

Psychoeducation on perfectionism will reduce symptoms of stress, anxiety, and depression compared to the healthy-habit and control groups.

Although the relationships among perfectionism, emotional regulation, and well-being are widely discussed in the literature, our research offers a new perspective by incorporating a two-stage research design that combines both cross-sectional and experimental approaches. Additionally, our hypotheses differentiate the impacts of two types of perfectionism—adaptive and maladaptive—on emotional regulation and well-being, allowing for a more detailed analysis of these relationships within the school environment. The novelty of our study lies in examining these variables not only in the context of students’ personal traits but also in relation to psychoeducational interventions aimed at modifying perfectionism and improving emotional well-being.

## 2. Methods and Study Group

### 2.1. Study Procedure

**Scheme 1:**
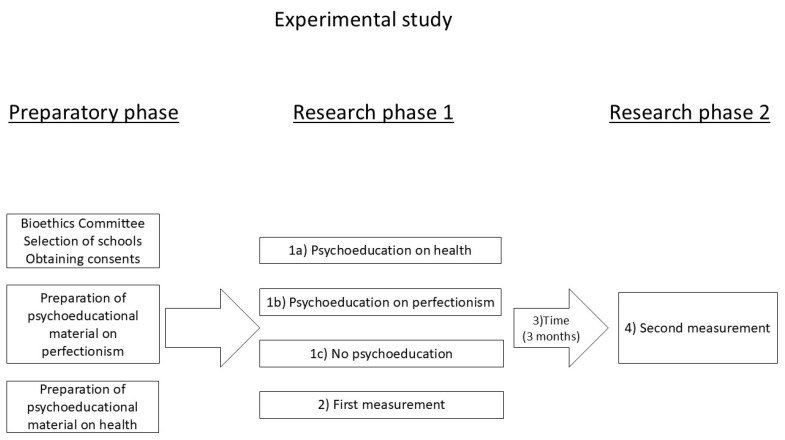
Study procedure for experimental study.

**Scheme 2:**
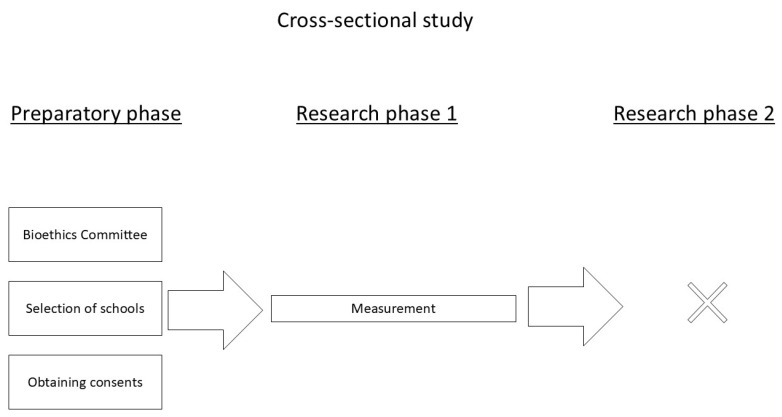
Study procedure for cross-sectional study.

To verify these assumptions, we conducted two experiments. The first study was cross-sectional. The second study was conducted naturally. The design of the overall study is presented in diagrams 1 and 2. Scheme 1 demonstrates the design of the experimental study, while Scheme 2 demonstrates the design of the cross-sectional study.

Before starting the study, the researchers obtained approval from the bioethics committee of their institution. Three high schools were selected for participation in the study. The selection of schools was based on the quality of education and the average performance of students. Schools were selected that were ranked high, medium, and low in order to maintain diversity, in line with the teenage population in Poland.

Students from each grade level (from grades 1 to 4) were selected from each school to participate in the study. Permission to conduct the survey was then obtained from school authorities, who chose the classes for the study without researcher intervention. Consent was obtained from the guardians of each participant, and those without consent were excluded. The preparation phase included developing psychoeducational materials, such as posters and workshop scenarios on cognitive–behavioural therapy techniques for perfectionism or healthy habits, created by psychologists and cognitive–behavioural psychotherapists. These materials covered education on the topic and potential causes, consequences, and strategies for addressing the phenomenon. The control group did not receive any interventions. In the cross-sectional survey, data were collected from preselected classes over 90 min. Participants could seek clarification, and research team members assisted. Participants were informed about the need for a smartphone with internet access, and all met this requirement. A QR code was provided to access a Google Form, including a participant ID to ensure anonymity and facilitate repeated measurements. The experimental study included a workshop on perfectionism or healthy habits, involving discussions, group work, and exercises, where participants gained theoretical and practical knowledge. The questionnaire was administered to participants before the workshop, while only the questionnaire was given to the control group.

No targeted interactions occurred during the three-month interval between research phases 1 and 2. Phase 2 involved only an experimental study. As in phase 1, a research team member administered the study via a Google Form, accessed by participants through a QR code scan.

Initially, 241 students participated in research phase 2, but some were excluded because of non-attendance in the second phase or incomplete questionnaires. Ultimately, 115 students were included in the statistical analysis. Because the survey was conducted on school premises, participation in the second phase depended on students’ attendance. Some missed this phase because of school absence, inability to attend, or other logistical issues.

### 2.2. Study Group

#### 2.2.1. Research Phase 1

A cross-sectional study (research phase 1) was conducted among 261 high school students: 154 females (59%) and 107 males (41%), aged from 14 to 18 (M = 17.14; SD = 1.33). Descriptive statistics indicated that 18% of the participants (*n* = 47) had a diagnosed mental illness or disorder, and 8.4% (*n* = 22) received pharmacological treatment and attended regular psychotherapy. Additional descriptive data for the study population are presented in Table 1.

#### 2.2.2. Research Phase 2

The experimental study (research phase 2) was conducted among *n* = 115 individuals divided into three subgroups—“healthy habits” (47 individuals, 40.9%), “perfectionism” (46 individuals, 40%), and the control group “control” (19.1%). The demographic data of the study’s subgroups and their statistical comparisons are summarised in Table 2.

### 2.3. Methods

An identical battery of tests was employed in both investigations. The investigation used a proprietary sociodemographic questionnaire to obtain the essential data necessary for characterising and describing the study cohort. Instruments designed to measure selected psychological characteristics were also employed.

(1) The Questionnaire of Adaptive and Maladaptive Perfectionism (KPAD), by K. Szczucka [57], contains 35 statements that fall on two scales. The PA scale’s score indicates the severity of the adaptive perfectionism, and the scale consists of 13 items. The PD scale’s score indicates the severity of the maladaptive perfectionism, and the scale consists of 22 items. The items are evaluated on a 7-point scale from 1—“strongly disagree” to 7—“strongly agree”. The questionnaire shows satisfactory psychometric properties. Cronbach’s α-values for the questionnaire scales were 0.896 for the adaptive perfectionism scale and 0.953 for the maladaptive perfectionism scale;
(2) The Depression, Stress, and Anxiety Scale (DASS-21), by P. Lovibond and Lovibond in a Polish adaptation by M. Makara-Studzińska et al. [58], consists of 21 items forming three scales: depression, anxiety, and stress. Each scale consists of 7 items. Subjects respond to them on a 4-point Likert-type scale from 0—“it didn’t apply to me at all” to 3—“it applied to me very much or most of the time”. Cronbach’s coefficients were 0.93 for the DASS-21 total score, 0.86 for the depression subscale, 0.84 for the anxiety subscale, and 0.85 for the DASS-21 stress subscale;
(3) The Difficulty in Emotional Regulation Scale (DERS), by K.L. Gratz and Loemer in a Polish adaptation by Dragan [59], consists of 36 statements forming six dimensions: disapproving emotional reactions (6 items), difficulty in engaging in goal-directed behaviour (5 items), difficulties in impulse control (6 items), deficiencies in awareness of emotions (6 items), limited access to emotional regulation strategies (8 items), and deficiencies in understanding emotions (5 items). Assessment of items is performed on a 5-point scale from 1—“almost never” to 5—“almost always”. The questionnaire is characterised by good overall psychometric properties and good reliability of the individual scales. The coefficient for the overall score was 0.92; for the disapproval subscale, 0.88; for the goal subscale, 0.88; for the impulse subscale, 0.75; for the awareness subscale, 0.89; for the strategy subscale, 0.94; and for the clarity subscale, 0.92.

### 2.4. Statistical Analyses

#### 2.4.1. Research 1

To verify the research hypotheses, statistical analyses were conducted utilising the IBM SPSS Statistics 25 package. The analyses encompassed descriptive statistics, with Shapiro–Wilk tests, Student’s *t*-tests for independent samples, Mann–Whitney U tests, Pearson’s r correlation analyses, and stepwise linear regression analyses. The conventional threshold of α = 0.05 was employed as the level of statistical significance.

#### 2.4.2. Research 2

To verify the research hypotheses, statistical analyses were conducted utilising the IBM SPSS Statistics 25 package. Employing this software, descriptive statistics analyses with Shapiro–Wilk tests, two-factor analyses of variance in a mixed design, and one-factor analyses of variance in a between-group design were performed. The level of statistical significance was established as the conventional threshold of α = 0.05.

## 3. Results

### 3.1. Research 1

#### 3.1.1. Basic Descriptive Statistics of Measured Quantitative Variables

In the initial phase of analysis, fundamental descriptive statistics were calculated for the quantitative variables under investigation. Shapiro–Wilk tests were subsequently conducted to assess the normality of the distributions of the analysed variables. As illustrated in Table 3, distributions approximating normality were observed for the levels of adaptive perfectionism and maladaptive perfectionism. The remaining variables exhibited distributions that deviated from the Gaussian distribution. In such circumstances, it is prudent to further examine the skewness and kurtosis values of the distributions of the variables under study. If these values fall within the range of ±2, it can be reasonably assumed that the distribution of the studied variable does not significantly deviate from symmetry with respect to the mean [60]. Such values were noted for both variables under investigation. Consequently, it was determined that statistical analyses would be conducted using parametric tests.

#### 3.1.2. Associations Between Perfectionism Levels and Symptoms of Depression, Anxiety, and Stress, and Emotional Regulation Difficulties

The subsequent analysis investigated the potential correlations between perfectionism levels and measures of depression, anxiety, and stress, and emotional regulation difficulties. Pearson’s r correlation analysis was performed. Several statistically significant correlations were observed (Table 4). The level of adaptive perfectionism was negatively correlated with the emotional understanding deficiency scale. This indicates that higher levels of adaptive perfectionism among participants were associated with lower levels of deficiencies in emotional understanding. The strength of this relationship is low. Conversely, the level of maladaptive perfectionism was positively correlated with depression, anxiety, stress, and various scales of emotional regulation difficulties, including non-acceptance of emotional reactions, difficulty in goal-directed behaviour implementation, difficulty in impulse control, limited access to emotional regulation strategies, and deficiencies in emotional understanding. Additionally, maladaptive perfectionism was negatively correlated with the scale of deficiencies in emotional awareness. These findings suggest that higher levels of maladaptive perfectionism are associated with lower levels of deficits in emotional awareness and higher levels of other dimensions of emotional regulation difficulties, as well as depression, anxiety, and stress. The strength of these correlations varied, ranging from low for the scale of difficulty in implementing goal-directed behaviour to moderately high for the scales of disapproval of emotional reactions, difficulty in impulse control, and deficits in emotional understanding and high for the remaining dimensions. Correlations that were not explicitly mentioned were not statistically significant.

#### 3.1.3. Associations Between Levels of Depression, Anxiety, and Stress, and Emotional Regulation Difficulties

The subsequent analysis investigated potential correlations between levels of depression, anxiety, and stress, and the degree of difficulty in emotional regulation. A series of Pearson’s r correlation analyses was conducted. As illustrated in Table 5, all the examined correlations were statistically significant. The levels of depression, anxiety, and stress demonstrated covariance, with lower levels for the Emotional Awareness Deficit Scale and higher levels for the other dimensions of emotional regulation difficulty. The strength of the correlations between anxiety and stress and the difficulty in implementing goal-directed behaviour scales was low. The correlations between depression, anxiety, and stress and the scale of the disapproval of emotional reactions, as well as between depression and stress and the scales of difficulty in impulse control and deficiencies in understanding emotions were moderately high. The correlations of the remaining relationships were characterised as high.

#### 3.1.4. Levels of Perfectionism, Depression, Anxiety, and Stress and Difficulties in Emotional Regulation vs. Students’ Diagnosis of Mental Diseases/Disorders

The subsequent analysis investigated whether the levels of the psychological variables under examination—perfectionism, depression, anxiety, stress, and difficulties in emotional regulation—varied according to students’ diagnoses of mental diseases/disorders. A series of Mann–Whitney U tests was conducted because of the substantial disparity in the sizes of the groups being compared. As shown in Table 6, eight significant differences were observed. Students diagnosed with mental illnesses/disorders exhibited higher levels of maladaptive perfectionism, depression, anxiety, stress, scales of non-acceptance of emotional reactions, limited access to emotional regulation strategies, and deficits in emotional understanding while demonstrating lower levels of deficits in emotional awareness. The magnitude of the observed effects, as measured by the r coefficient, was moderately high for depression and low for the other variables. The remaining differences, which were not mentioned above, were not statistically significant.

### 3.2. Research 2

#### 3.2.1. Basic Descriptive Statistics of the Measured Quantitative Variables

In the initial phase of this study, basic descriptive statistics of the quantitative variables were calculated. Shapiro–Wilk tests were subsequently conducted to assess the normality of the distributions of the analysed variables. These analyses were performed for Measures I and II. As indicated in Table 7, distributions approximating normality were observed for levels of adaptive perfectionism; maladaptive perfectionism in both measures; non-acceptance of emotional reactions and deficits in understanding of emotions in Measure I; deficits in awareness of emotions in Measure II; and depression, anxiety, stress, scales of non-acceptance of emotional reactions, difficulties in implementing goal-directed behaviour, deficits in awareness of emotions, limited access to emotional regulation strategies, and deficits in understanding of emotions for differences between measures. The other variables exhibited distributions that deviated from a Gaussian distribution. In such instances, it is prudent to further examine the skewness and kurtosis values of the distributions of the variables under study. If these values fall within the range of ±2, it can be reasonably assumed that the distribution of the studied variable does not significantly deviate from symmetry with respect to the mean [60]. These values were observed for both variables under investigation. Consequently, it was determined that statistical analyses would be conducted using parametric tests.

#### 3.2.2. Change in the Level of Studied Variables Between Measure I and Measure II in the Study Groups

In the next step, we investigated whether the level of the studied psychological variables was different in the study samples in Measure I and Measure II. Thus, a series of two-factor analyses of variance was performed using a mixed scheme. The interaction effect and simple effects were interpreted because the main effects of the study group membership (by averaging the level of the studied traits in both measures) and the moment of the measure (by averaging the level of the studied traits in the three study groups) had no interpretive significance in this study.

No statistically significant interaction effect of the studied factors was observed, F(2, 112) = 0.19; *p* = 0.831; η^2^ = 0. Nevertheless, a simple effect analysis was conducted. A statistically significant change in the level of the trait under study between Measures I and II was found in the healthy-habit group, F(1, 112) = 12.62; *p* = 0.001; η^2^ = 0.10, and in the perfectionism group, F(1, 112) = 7.22; *p* = 0.008; η^2^ = 0.06. A decrease in the level of the trait under study was observed in both groups, with the change in the former being approximately twice as large. However, the difference in the control group was not statistically significant, F(1, 112) = 3.90; *p* = 0.051; η^2^ = 0.03. Furthermore, the difference between the compared groups was not statistically significant for either Measure I, F(2, 112) = 0.27; *p* = 0.764; η^2^ = 0.01; or Measure II, F(2, 112) = 0.33; *p* = 0.719; η^2^ = 0.01.

These results indicated no statistically significant interaction effects among the factors under investigation, F(2, 112) = 1.29; *p* = 0.281; η^2^ = 0.02. Despite this finding, a simple effect analysis was conducted. The analysis revealed no statistically significant change in the level of the studied trait between Measures I and II in the healthy-habit group, F(1, 112) = 0.02; *p* = 0.894; η^2^ = 0; the perfectionism group, F(1, 112) = 3.47; *p* = 0.065; η^2^ = 0.03; or the questionnaire group, F(1, 112) = 2.02; *p* = 0.158; η^2^ = 0.02. Furthermore, the differences between the groups did not reach statistical significance in either Measure I, F(2, 112) = 1.60; *p* = 0.207; η^2^ = 0.03; or Measure II, F(2, 112) = 2.24; *p* = 0.111; η^2^ = 0.04.

All the results described above are presented collectively in Table 8.

The interaction effect of the studied factors was not statistically significant, F(2, 112) = 0.97; *p* = 0.384; η^2^ = 0.02. Nevertheless, a simple effect analysis was conducted. A statistically significant change in the level of the trait under investigation was observed between Measures I and II in the healthy-habit group, F(1, 112) = 5.66; *p* = 0.019; η^2^ = 0.05 and in the perfectionism group, F(1, 112) = 4.25; *p* = 0.042; η^2^ = 0.04. An increase in the level of the trait under investigation was recorded in both groups, with the change in the former being marginally greater. However, the difference in the control group was not statistically significant, F(1, 112) = 0; *p* = 1; η^2^ = 0. In contrast, the difference between the compared groups was statistically significant for Measure II, F(2, 112) = 4.13; *p* = 0.025; η^2^ = 0.07. Consequently, post hoc analyses were performed using the Sidak test. Statistically significant differences were observed. Stress levels were higher in the healthy-habit group than in the control group (*p* = 0.019). The perfectionism group did not differ significantly from the healthy-habit (*p* = 0.938) and control (*p* = 0.057) groups. In Measure I, the differences between groups were not statistically significant, F(2, 112) = 1.73; *p* = 0.183; η^2^ = 0.03). Table 9 presents these results.

## 4. Discussion

The purposes of Study 1 were to examine the relationships among perfectionism (adaptive and maladaptive), factors related to mental health (anxiety, stress, and depression), and emotional regulation among adolescents and to identify predictors of adaptive and maladaptive perfectionism among students. The findings of the present study offer significant insights into the intricate interplay between different forms of perfectionism and their correlations with emotional intelligence and mental health factors. Adaptive perfectionism, typified by elevated personal standards and the necessity for effective organisation, exhibited a negative correlation with the extent of deficits in emotional understanding. This finding suggests that individuals with adaptive perfectionism may have an advantage in identifying and interpreting their own emotions, which, in turn, may support more effective emotional regulation strategies. A more profound understanding of emotions could also act as a protective mechanism against the adverse consequences of stress and pressure to achieve high standards, thereby enhancing psychological well-being.

Conversely, maladaptive perfectionism, characterised by an excessive focus on high expectations, frequent criticism, and the attribution of undue importance to mistakes, has been shown to correlate positively with depression, anxiety, and stress. These findings indicate that individuals with maladaptive perfectionism are more likely to experience negative emotional and psychological outcomes, such as chronic feelings of inadequacy, low self-esteem, or ruminative tendencies. Such perfectionism can lead to emotional exhaustion and increase the risk of developing psychological disorders, such as depression or anxiety disorders. The results of studies conducted by other researchers are consistent with these findings. Maladaptive perfectionism has been shown to be associated with adverse outcomes, such as increased levels of negative emotions [61] and depression [62]. The results of previous studies show that maladaptive perfectionism not only makes people more susceptible to mental disorders but also makes diagnosed mental disorders more difficult to overcome when they occur [63]. Conversely, adaptive perfectionism, which motivates activity, serves as a protective factor against the development of depression [13]. Maladaptive perfectionism is associated with higher levels of perceived anxiety [64] and stress, particularly those associated with evaluation by others. No similar relationship was observed with adaptive perfectionism [65], suggesting its protective role in this regard. Specifically, the concept of adaptive perfectionism, which is characterised by elevated personal standards, a sense of accomplishment, and a focus on growth rather than the fear of failure, has been demonstrated to act as a buffer against the adverse effects of stress and anxiety [66]. Difficulties in emotional regulation, such as the non-acceptance of emotional reactions, difficulties in implementing goal-directed behaviour, difficulties in impulse control, limited access to emotional regulation strategies, and deficits in understanding emotions, correlate positively with maladaptive perfectionism. This indicates that individuals who pay excessive attention to their mistakes and focus more on criticism from others may manifest inappropriate strategies for managing emotions. Specifically, the inability to accept emotional responses may lead to heightened self-criticism and ruminative thought patterns, which can further exacerbate stress and negative emotional states. The limited access to effective emotional regulation strategies implies that these individuals often lack the tools to manage and alleviate emotional distress, leading to a vicious cycle where unaddressed emotions intensify perfectionistic fears. A further issue is the limited understanding of emotions, which may be because of deficits in emotional intelligence. This may prevent individuals from identifying triggers and seeking appropriate support or interventions. Furthermore, the challenges experienced in the implementation of goal-directed behaviour suggest that individuals diagnosed with maladaptive perfectionism may encounter difficulties in maintaining concentration and motivation in the presence of emotional distress, which may, consequently, lead to procrastination or avoidance behaviours. Impulse control challenges are also highlighted, with a tendency to react excessively or inappropriately under pressure. This may strain interpersonal relationships and reinforce feelings of inadequacy.

These results allow for the positive verification of Hypothesis 2. Global studies have demonstrated that adaptive perfectionism predicts a relative increase in adolescents’ use of reappraisal (i.e., an adaptive emotional regulation strategy) and a relative decrease in their non-acceptance of negative emotional reactions (i.e., difficulties in emotional regulation) [67]. Conversely, maladaptive perfectionism predicts a relative increase in adolescents’ difficulties in emotional regulation (i.e., a lack of acceptance of negative emotional reactions, difficulty in controlling impulsive behaviour when stressed, limited access to effective emotional regulation strategies, and a lack of emotional transparency) [61,67]. The results of other studies also suggest that ruminations, which are characterised by repetitive and unproductive thoughts, are a prevalent symptom of emotional regulation difficulties. These ruminations are often exacerbated by high levels of perfectionistic anxiety or non-adaptive perfectionism, leading to a substantial increase in perceived stress and anxiety [15]. The results of the present study indicated that higher levels of depression, anxiety, and stress are associated with increased difficulties in emotional regulation. This demonstrates the relevance and interconnectedness of these psychological factors and their potential impacts on adolescents’ mental health and emotional well-being. The presence of emotional regulation difficulties, including a lack of acceptance of one’s own emotional reactions, challenges in managing behaviour in the face of emotions, limited ability to control impulses, a lack of access to effective regulatory strategies, and deficits in understanding emotions, can further compound the negative effects associated with elevated levels of depression, anxiety, and stress. Depression, anxiety, and stress not only serve to reinforce difficulties in regulating emotions but also can act as triggers for unhealthy emotional coping patterns, such as ruminations, problem avoidance, or negative re-evaluation of situations. Such interactions can lead to impaired psychological functioning, lower self-esteem, and difficulties in daily life, especially in the context of the challenges of adolescence. This allowed for the positive verification of Hypothesis 3. Emotional regulation skills develop during adolescence. Adolescence is also a period of risk for anxiety and depressive disorders [68]. The results of global research corroborate the findings of the present study. The significant role of emotional regulation in the development and maintenance of internalising disorders has been previously demonstrated. Maladaptive emotional coping strategies are associated with higher levels of depression [69] and perceived high levels of anxiety and anxiety disorders [69,70].

The purpose of Study 2 was to determine the effectiveness of perfectionism psychoeducation and healthy habits in altering the severity of maladaptive and adaptive perfectionism and associated symptoms, such as depression, anxiety, and stress, and to ascertain differences in the effectiveness of perfectionism psychoeducation and healthy habits. The results indicated no significant alterations in the severity of adaptive perfectionism in adolescents, following the implementation of the intervention (psychoeducation), in any of the three study groups. Adaptive perfectionism is associated with high self-esteem [71], motivation to establish and pursue realistic goals, and success-oriented work [72]. Both the experimental and control groups demonstrated no statistically significant changes in the severity of adaptive perfectionism. This may suggest that following the introduction of the intervention, subjects with high levels of adaptive perfectionism, who did not experience negative consequences of their actions, did not implement changes, resulting in the absence of significant differences in the severity of the aforementioned variable. It is also conceivable that the psychoeducational methods employed did not adequately emphasise the cultivation of adaptive perfectionism traits, such as flexibility in achieving goals or acceptance of mistakes. This could have fostered the positive facets of this form of perfectionism. Additionally, the absence of observed change may be attributed to the notion that adaptive perfectionism constitutes a more stable personality trait, less susceptible to the immediate impact of short-term interventions. This observation underscores the necessity for the development of more comprehensive and multifaceted support programmes in subsequent research endeavours.

These results do not support Hypothesis 4, but they constitute an important starting point for further considerations. In future research, the development of interventions that target both a reduction in maladaptive perfectionism and the enhancement of adaptive facets should be a priority, with the aim of providing young individuals with enhanced outcomes in both educational and emotional domains. Significant decreases in the level of maladaptive perfectionism were observed in the group that participated in psychoeducation on healthy habits and the group that received psychoeducation on perfectionism. A more substantial change was observed in the healthy-habit group. This suggests that interventions focusing on promoting healthy habits and addressing the consequences of perfectionistic fears may be particularly effective in reducing harmful perfectionist tendencies that threaten mental health. The enhancement of awareness concerning destructive thought patterns and behaviours associated with perfectionism among participants may be ascribed to the integration of healthy habits and psychoeducation about perfectionism. It is hypothesised that the healthy-habit group may have experienced a more significant decline in maladaptive perfectionism because of the more general and practical tips that were more readily applicable to everyday life. The practical strategies employed in the workshops, which focused on time management, rest, and the cultivation of healthier relationships, may have contributed to reductions in stress and anxiety, which are known to fuel maladaptive perfectionistic tendencies. The psychoeducational intervention, despite its specific focus on addressing maladaptive perfectionism, may have yielded less favourable outcomes during the observed period because of the multifaceted nature of the changes required, encompassing not only daily but also cognitive functioning. It is also conceivable that the created psychoeducational materials on perfectionism did not constitute a sufficient resource for better coping with maladaptive perfectionism and influenced the participants at a similar level as the workshops that encouraged them to improve their mental life by introducing healthy habits in functioning and thinking strategies. These results partially confirm Hypothesis 5 and indicated significant decreases in depression levels across all three groups, with the most pronounced effect observed in the group that participated in perfectionism classes. This finding suggests that targeted perfectionism interventions may have a beneficial effect on reducing depressive symptoms. These results may be attributed to a reduction in maladaptive perfectionism, following the introduction of psychoeducation, which may have indirectly led to decreases in depression levels among the adolescents studied. The findings of other studies also support this conclusion. Research has demonstrated that reducing perfectionist anxiety can significantly alleviate depressive symptoms [73,74]. Psychoeducation related to healthy habits included, among others, topics of sleep, nutrition, physical activity, and the negative consequences of incorrect habits, including those related to mental health. Depressive symptoms include, in addition to low mood, difficulty in sleeping and reduced psychophysical activity. Psychoeducation regarding healthy habits could reduce depression in the respondents because of their knowledge about the importance of proper functioning. No significant changes were observed in anxiety levels across the groups, indicating that the interventions may not have been as effective in addressing anxiety. It is noteworthy that the interventions (psychoeducation) implemented in the experimental groups did not specifically target anxiety. Furthermore, anxiety may be considered as a permanent feature of an individual.

Stress levels increased significantly in both experimental groups, with a slightly greater increase observed in the group that participated in psychoeducation related to healthy habits. However, no significant changes were observed in the control group. This finding suggests that interventions targeting healthy habits and perfectionism may have unintended consequences, potentially leading to increases in stress levels, and that acquiring new knowledge about perfectionism and healthy habits may serve as a stressor for adolescents who have not developed adaptive stress-coping mechanisms. The heightened stress levels observed in the experimental groups may be attributable to an enhanced sense of self-awareness concerning their deficiencies and the necessity to implement modifications to their present functioning. The acknowledgement that certain habitual coping mechanisms or stress management strategies may be ineffective, or even detrimental, can lead to a transient escalation in emotional tension. These results partially confirm Hypothesis 6, with partial confirmation derived from the observed decreases in depression levels in the group receiving psychoeducation on perfectionism. The findings of the present study demonstrate the complex relationships among perfectionism, mental health, and emotional regulation among adolescents. These results indicate that interventions targeting healthy habits and perfectionism may have positive effects on maladaptive perfectionism and levels of depression. However, the observed increases in stress levels underscore the necessity of carefully considering and monitoring potential adverse effects when implementing such interventions. Future research should focus on further analysis of the mechanisms leading to increases in stress levels following interventions. It is imperative to ascertain whether the heightened stress experienced is a transient phenomenon resulting from adaptation to novel strategies and knowledge or whether it necessitates supplementary support, such as training in stress-coping skills. Furthermore, the development of interventions incorporating stress reduction techniques, such as relaxation training, mindfulness, or cognitive–behavioural strategies, is recommended to mitigate the risk of escalating emotional tension during the implementation of such programmes.

## 5. Conclusions

### 5.1. Conclusions of This Study

The results of Study 1 demonstrated positive correlations between maladaptive perfectionism and depression, anxiety, stress, and various dimensions of emotional regulation difficulties. Conversely, adaptive perfectionism was negatively correlated with a deficiency in understanding emotions. Participants diagnosed with a mental disorder displayed higher levels of maladaptive perfectionism. Based on these findings, it can be inferred that higher levels of maladaptive perfectionism are associated with an increased likelihood of developing mental disorders and greater difficulty in regulating emotions in adolescents. Consequently, it can be concluded that in individuals with high levels of perfectionism, the adaptive form may serve as a protective factor. The results of Study 2 revealed no significant alterations in adaptive perfectionism across the groups. However, with regard to maladaptive perfectionism, significant changes were observed, specifically, a decrease in the severity of this type of perfectionism among the group of students who received the intervention (psychoeducation—healthy habits and perfectionism). Comparable results were noted for the level of depression, with a reduction in the severity of this variable in groups following psychoeducation. These findings suggest that targeted interventions can be effective in diminishing maladaptive perfectionism and its associated negative effects. The development and implementation of tailored interventions that address perfectionism, emotional regulation skills, and healthy habits may contribute to enhanced mental health and school performance among students. Such interventions can assist students in establishing more realistic goals and in developing effective strategies for managing emotions and stress.

### 5.2. Innovation of This Method and Contribution of This Study

The proposed research method, encompassing two experimental stages, stands out in comparison to previous studies because of its combination of a two-stage research design with the use of various psychometric tools and psychoeducational interventions. Unlike earlier studies that focused on a single approach for analysing perfectionistic or emotional behaviours, our method integrates these aspects in the context of psychological variables, such as depression, anxiety, stress, and emotional regulation. The use of two distinct experiments, one cross-sectional and the other experimental, allows for a deeper analysis of the variability in the phenomena under investigation in different contexts.

In the first study (cross-sectional), data were collected from students in preselected classes, which ensured a representative sample, reflecting the normal distribution of school achievement in the study group. In contrast, the second study (experimental) introduced a psychoeducational intervention aimed at familiarising participants with the topics of perfectionism or healthy habits, which represented a unique contribution to the study of the impacts of psychological education on emotional regulation and reductions in perfectionism levels.

The motivation for conducting this study stemmed from the need for a more in-depth analysis of the impact of perfectionism on emotional regulation, particularly in the context of psychoeducational interventions. Although previous research has focused on individual aspects, our methodology integrates diverse approaches, enabling a better understanding of the interrelationships between the variables under study. The introduction of educational interventions, both in the context of perfectionism and healthy habits, provides an opportunity to assess the effectiveness of such actions in reducing emotional issues.

The contribution of this study to the psychological literature is twofold. First, it provides new empirical data on the impacts of perfectionism and emotions on individual behaviour. Second, it makes a valuable methodological contribution by offering an innovative integration of psychoeducational interventions with traditional psychometric tools. This approach not only allows for a more accurate assessment of individual differences in emotions and perfectionism but also opens up new possibilities for designing therapeutic interventions.

### 5.3. Implications and Future Studies

Subsequent research should concentrate on the longitudinal effects of such interventions and their potential to enhance student well-being. It would also be important to emphasise the relevance of individual differences, such as temperament, which can be associated with both perfectionism and mental health, as well as affect the effectiveness of psychoeducation and other interventions. Furthermore, it is imperative to investigate the possibility of identifying specific components of interventions that demonstrate the highest efficacy, thereby facilitating the refinement and optimisation of future programmes. This study reports on the short-term effects of the interventions. Future research should focus on long-term impacts (six months, a year, etc.). That way, the scope of future research could also include the long-term effects of the intervention on academic performance, peer relationships, and other psychosocial factors. In addition, the above study focused on one-time workshops on perfectionism and healthy habits. It would be worthwhile, in the future, to create a programme that would consist of a series of several workshops conducted by specialists, which could strengthen the effects of psychoeducation. Little effect was observed in the above study, making the one-time workshops a limitation of the study. It would also be worthwhile to verify which cognitive–behavioural techniques (e.g., reformulation of maladaptive thoughts and stress management) are most effective in reducing maladaptive perfectionism.

### 5.4. Limitations

This research study’s limitations encompass the restricted participant pool and the consequent challenges in generalising the findings to a broader demographic. In a comparative analysis of dual measures, the investigators were unable to control for potential confounding variables that may have influenced temporal variations in the examined parameters. Moreover, this study’s reliance on self-reported survey instruments introduces a potential element of subjectivity in assessing the magnitude of the variables under investigation. An additional limitation of this study was that it focused on intergroup comparisons and did not consider individual differences in response to the intervention. Although sociodemographic data and mental health variables were collected, the study design and statistical methods limited the examination of individual trajectories or differential outcomes.

## Figures and Tables

**Table 1:** Sociodemographic data—research phase 1.

		*n*	%
Gender	Woman	143	54.8
	Man	102	39.1
	Other	16	6.1
Size of the place of residence	Village	54	20.7
	Town with <50,000 inhabitants	32	12.3
	City with 50,000–100,000 inhabitants	4	1.5
	City with 100,000–500,000 inhabitants	167	64
	City with >500 thousand inhabitants	4	1.5
Diagnosed mental health issue	Yes	47	18
	No	214	82
Pharmacotherapy	Yes	22	8.4
	No	239	91.6
Psychotherapy	Yes	22	8.4
	No	239	91.6
Previous psychoeducation (perfectionism and healthy habits)	Yes	137	52.5
	No	124	47.5

**Table 2:** Sociodemographic data—research phase 2.

			Healthy Habits	Perfectionism	Control	
Gender	Woman	*n*	33	41	19	χ^2^(2) = 5.91; *p* = 0.052
		%	70.20%	89.10%	86.40%	
	Man	*n*	14	5	3	
		%	29.80%	10.90%	13.60%	
Age	M (SD)		17.59 (0.83)	15.65 (1.06)	16.27 (0.55)	F(2, 111) = 55.68 *p* < 0.001
	Min–Max		15–19	14–19	15–17	
Size of the place of residence	Town with <50,000 inhabitants	*n*	6	3	0	
		%	12.80%	6.50%	0.00%	
	City with 50,000–100,000 inhabitants	*n*	2	0	0	
		%	4.30%	0.00%	0.00%	
	City with 100,000–500,000 inhabitants	*n*	30	27	18	
		%	63.80%	58.70%	81.80%	
	City with >500 thousand inhabitants	*n*	0	0	1	
		%	0.00%	0.00%	4.50%	
Diagnosed mental health issue	Yes	*n*	7	7	3	χ^2^(2) = 0.03; *p* = 0.985
		%	14.90%	15.20%	13.60%	
	No	*n*	40	39	19	
		%	85.10%	84.80%	86.40%	
Psychiatric treatment	Yes	*n*	1	7	2	Exact Fisher’s Test *p* = 0.065
		%	2.10%	15.20%	9.10%	
	No	*n*	46	39	20	
		%	97.90%	84.80%	90.90%	
Psychotherapy	Yes	*n*	1	3	0	Exact Fisher’s Test *p* = 0.414
		%	2.10%	6.50%	0.00%	
	No	*n*	46	43	22	
		%	97.90%	93.50%	100.00%	
Average marks	M (SD)		3.78 (0.71)	4.31 (0.90)	3.74 (0.61)	F(2, 111) = 6.73; *p* = 0.002
	Min–Max		3.00–5.30	2.00–5.40	2.00–5.00	

**Table 3:** Descriptive statistics of the quantitative variables under study.

Variable	M	Me	SD	Sk.	Kurt.	Min.	Max.	D	*p*
Adaptive perfectionism	59.63	59	13.56	0.06	−0.39	23	89	0.99	0.155
Maladaptive perfectionism	87.58	87.50	23.99	0.06	−0.47	32	143	0.99	0.414
Depression	19.40	18	9.55	0.52	−0.51	4	46	0.96	<0.001
Anxiety	15.21	14	10.67	0.67	−0.53	0	42	0.93	<0.001
Stress	15.57	14	10.66	0.53	−0.62	0	42	0.95	<0.001
Non-acceptance of emotional reactions	16.55	17	3.85	0.23	−0.13	6	26	0.98	0.024
Difficulty in goal-directed behaviour implementation	13.13	12	3.78	0.82	0.12	6	25	0.93	<0.001
Difficulty in impulse control	15.72	15	3.24	−0.08	2.57	0	25	0.96	<0.001
Deficits in awareness of emotions	20.25	21	4.29	−0.40	−0.46	9	30	0.97	<0.001
Limited access to emotional regulation strategies	21.29	20	4.92	0.48	−0.37	12	35	0.97	<0.001
Deficiencies in emotional understanding	14.71	14.50	2.62	0.40	0.03	8	22	0.97	<0.001

Annotations: M—mean; Me—median; SD—standard deviation; Sk.—skewness; Kurt.—kurtosis; Min and Max.—lowest and highest values of the distribution; D—Shapiro–Wilk test result; *p*—significance.

**Table 4:** Relationships between levels of perfectionism and levels of depression, anxiety, and stress, and difficulties in regulating emotions.

	Adaptive Perfectionism	Maladaptive Perfectionism
Depression	0.08	0.64 ***
Anxiety	0	0.66 ***
Stress	0.01	0.65 ***
Non-acceptance of emotional reactions	0.05	0.37 ***
Difficulty in goal-directed behaviour implementation	−0.11	0.25 ***
Difficulty in impulse control	−0.12	0.40 ***
Deficits in awareness of emotions	0.11	−0.52 ***
Limited access to emotional regulation strategies	−0.14	0.50 ***
Deficiencies in emotional understanding	−0.21 **	0.45 ***

Annotations: **—*p* < 0.01; ***—*p* < 0.001.

**Table 5:** Relationships between levels of depression, anxiety, and stress and the level of difficulty in regulating emotions.

	Depression	Anxiety	Stress
Non-acceptance of emotional reactions	0.42 ***	0.36 ***	0.41 ***
Difficulty in goal-directed behaviour implementation	0.43 ***	0.27 ***	0.27 ***
Difficulty in impulse control	0.48 ***	0.52 ***	0.45 ***
Deficits in awareness of emotions	−0.61 ***	−0.56 ***	−0.57 ***
Limited access to emotional regulation strategies	0.60 ***	0.55 ***	0.55 ***
Deficiencies in emotional understanding	0.40 ***	0.56 ***	0.51 ***

Annotations: ***—*p* < 0.001.

**Table 6:** Levels of perfectionism, depression, anxiety, and stress and difficulties in regulating emotions vs. students’ mental illness/disorder diagnosis.

	Yes (*n* = 24)		No (*n* = 170)					
	M	SD	M	SD	U	Z	*p*	r
Adaptive perfectionism	58.79	12.83	59.75	13.69	1975.0	−0.25	0.801	0.02
Maladaptive perfectionism	102.92	21.33	85.42	23.61	1164.5	−3.40	0.001	0.24
Depression	25.92	9.01	18.48	9.29	1119.5	−3.58	<0.001	0.26
Anxiety	21.75	12.46	14.28	10.10	1304.5	−2.86	0.004	0.21
Stress	22.42	12.61	14.60	10.03	1317.0	−2.81	0.005	0.20
Non-acceptance of emotional reactions	18.75	4.78	16.24	3.61	1391.0	−2.53	0.011	0.18
Difficulty in goal-directed behaviour implementation	13.96	4.23	13.01	3.71	1775.5	−1.03	0.302	0.07
Difficulty in impulse control	16.56	5.07	15.60	2.88	1769.0	−1.36	0.174	0.10
Deficits in awareness of emotions	17.63	4.36	20.62	4.16	1247.5	−3.09	0.002	0.22
Limited access to emotional regulation strategies	24.75	5.87	20.81	4.59	1249.5	−3.08	0.002	0.22
Deficiencies in emotional understanding	15.88	2.85	14.55	2.55	1450.0	−2.31	0.021	0.17

Annotations: M—mean; SD—standard deviation; U—Mann–Whitney U test result; Z—standardised score; *p*—significance; r—coefficient of effect strength.

**Table 7:** Basic descriptive statistics of the quantitative variables under study.

Variable	M	Me	SD	Sk.	Kurt.	Min.	Max.	D	*p*
Measure I									
Adaptive perfectionism	58.50	58	12.40	0.19	−0.12	23	86	0.99	0.333
Maladaptive perfectionism	92.87	93	24.10	−0.09	−0.52	32	143	0.99	0.556
Depression	21.50	22	9.91	0.30	−0.77	6	46	0.97	0.005
Anxiety	16.83	14	10.80	0.61	−0.72	2	42	0.93	<0.001
Stress	17.97	16	10.91	0.28	−0.87	0	42	0.96	0.003
Non-acceptance of emotional reactions	16.95	17	3.87	0.11	−0.39	7	26	0.99	0.324
Difficulty in goal-directed behaviour implementation	13.68	13	4.03	0.69	−0.22	7	25	0.94	<0.001
Difficulty in impulse control	16.13	16	3.34	0.53	0.03	9	25	0.97	0.007
Deficits in awareness of emotions	19.73	20	4.36	−0.44	−0.67	9	27	0.96	0.001
Limited access to emotional regulation strategies	22.16	22	5.05	0.50	−0.56	13	35	0.96	0.002
Deficiencies in emotional understanding	14.91	15	2.62	0.29	0.09	8	22	0.98	0.072
Measure II									
Adaptive perfectionism	58.93	59	12.44	−0.03	0.08	25	91	0.99	0.923
Maladaptive perfectionism	85.37	85	25.81	0.05	−0.29	23	138	0.99	0.407
Depression	17.35	16	11.95	0.28	−1.09	0	40	0.94	<0.001
Anxiety	15.44	14	11.73	0.46	−0.84	0	42	0.94	<0.001
Stress	20.45	22	11.89	−0.02	−1.19	0	42	0.95	<0.001
Non-acceptance of emotional reactions	14.03	13	6.62	0.61	−0.61	6	30	0.93	<0.001
Difficulty in goal-directed behaviour implementation	16.27	16	5.08	0	−0.94	6	25	0.97	0.006
Difficulty in impulse control	15.27	14	6.40	0.37	−0.79	6	30	0.96	0.001
Deficits in awareness of emotions	19.12	19	4.73	−0.05	−0.25	6	30	0.99	0.462
Limited access to emotional regulation strategies	21.08	21	7.45	0.19	−0.88	8	39	0.97	0.023
Deficiencies in emotional understanding	14.53	14	4.83	0.29	−0.81	6	25	0.97	0.007

Annotations: M—mean; Me—median; SD—standard deviation; Sk.—skewness; Kurt.—kurtosis; Min and Max.—lowest and highest values of the distribution; D—Shapiro–Wilk test result; *p*—significance.

**Table 8:** Levels of adaptative and maladaptive perfectionism, depression, anxiety, and stress in the study groups in Measure I and Measure II.

		Healthy Habits		Perfectionism		Control	
Measure		I	II	I	II	I	II
Adaptive perfectionism	M	58.87	60.06	58.11	58.07	58.50	58.32
	SD	13.52	13.32	11.77	10.72	11.71	14.16
Maladaptive perfectionism	M	93.04	84.43	94.24	87.65	89.64	82.64
	SD	25.30	26.58	22.45	21.98	25.58	31.83
Depression	M	21.91	18.35	22.61	18.09	18.91	13.73
	SD	9.20	11.32	10.66	12.83	9.48	11.10
Anxiety	M	16.85	17.02	18.43	16.04	13.45	10.82
	SD	9.79	11.23	12.34	12.76	8.93	9.67
Stress	M	19.28	22.55	18.43	21.30	14.18	14.18
	SD	9.78	11.09	12.01	11.86	10.42	14.18

**Table 9:** Comparison of changes in the characteristics studied in the three research groups.

	Healthy Habits (*n* = 47)		Perfectionism (*n* = 46)		Control (*n* = 22)				
	M	SD	M	SD	M	SD	F	*p*	η^2^
Adaptive perfectionism	−1.19	9.29	0.04	8.85	0.18	9.65	0.27	0.762	0
Maladaptive perfectionism	8.62	19.02	6.59	14.29	7.00	15.64	0.19	0.831	0
Depression	3.57	11.02	4.52	11.01	5.18	9.43	0.19	0.826	0.00
Anxiety	−0.17	9.61	2.39	7.31	2.64	9.35	1.28	0.280	0.02
Stress	−3.28	9.19	−2.87	9.57	0.00	9.70	0.97	0.384	0.02

Annotations: M—mean; SD—standard deviation; F—ANOVA test result; *p*—significance; η^2^—coefficient of effect strength. Positive value—the score in Measure I was higher than that in Measure II.

## Data Availability

The raw data supporting the conclusions of this article will be made available by the authors on request because of the sensitivity of the data collected during the study.

## References

[1] Pearlman-Avnion S., Harduf R. (2019). Procrastination, perfectionism, and locus of control in academic settings. Szk. Spec..

[2] Frost R.O., Marten P., Lahart C., Rosenblate R. (1990). The dimensions of perfectionism. Cogn. Ther. Res..

[3] Sanecka E. (2020). Perfekcjonizm—Pozytywny czy negatywny—Adaptacyjne i dezadaptacyjne formy perfekcjonizmu. Ogrody Nauk I Szt..

[4] Włodarczyk D., Obacz W. (2013). Perfectionism, selected demographic and job characteristics as predictors of burnout in operating suite nurses. Medycyna Pracy.

[5] Bieling P.J., Israeli A., Smith J., Antony M.M. (2003). Making the grade: The behavioural consequences of perfectionism in the classroom. Personal. Individ. Differ..

[6] Rice K.G., Richardson C.M., Ray M.E., Sirois F.M., Molnar D.S. (2016). Perfectionism in academic settings. Perfectionism, Health, and Well-Being.

[7] Speirs Neumeister K.L., Williams K.K., Cross T.L. (2009). Gifted high-school students’ perspectives on the development of perfectionism. Roeper Rev..

[8] Neumeister K.L.S. (2004). Understanding the relationship between perfectionism and achievement motivation in gifted college students. Gift. Child Q..

[9] Madigan D.J. (2019). A meta-analysis of perfectionism and academic achievement. Educ. Psychol. Rev..

[10] Vicent M., Gonzálvez C., Sanmartín R., Fernández-Sogorb A., Cargua-García N.I., García-Fernández J.M. (2019). Perfectionism and school anxiety: More evidence about the 2 × 2 model of perfectionism in an Ecuadorian population. Sch. Psychol. Int..

[11] Abdollahi A., Carlbring P., Vaez E. (2018). Perfectionism and test anxiety among high-school students: The moderating role of academic hardiness. Curr. Psychol..

[12] Affrunti N.W., Woodruff-Borden J., Stoeber J. (2018). Perfectionism and anxiety in children. The Psychology of Perfectionism: Theory, Research, Applications.

[13] Chai L., Yang W., Zhang J., Chen S., Hennessy D.A., Liu Y. (2019). Relationship between perfectionism and depression among Chinese college students with self-esteem as a mediator. OMEGA—J. Death Dying.

[14] Callaghan T., Greene D., Shafran R., Lunn J., Egan S.J. (2023). The relationships between perfectionism and symptoms of depression, anxiety and obsessive-compulsive disorder in adults: A systematic review and meta-analysis. Cogn. Behav. Ther..

[15] Kahn J.H., Woodrum J.L., Han S. (2021). Perfectionistic concerns, emotion regulation, and psychological distress: Competing predictors and indirect effects. Couns. Psychol. Q..

[16] Huang I., Short M.A., Bartel K., O’Shea A., Hiller R.M., Lovato N., Micic G., Oliver M., Gradisar M. (2020). The roles of repetitive negative thinking and perfectionism in explaining the relationship between sleep onset difficulties and depressed mood in adolescents. Sleep Health.

[17] Damian L.E., Stoeber J., Negru-Subtirica O., Băban A. (2017). Perfectionism and school engagement: A three-wave longitudinal study. Pers. Individ. Differ..

[18] Bas A.U. (2011). Dimensions of perfectionism in elementary school-aged children: Associations with anxiety, life satisfaction, academic achievement. Egit. Bilim-Educ. Sci..

[19] Curran T., Hill A.P. (2019). Perfectionism is increasing over time: A meta-analysis of birth cohort differences from 1989 to 2016. Psychol. Bull..

[20] Sand L., Bøe T., Shafran R., Stormark K.M., Hysing M. (2021). Perfectionism in adolescence: Associations with gender, age, and socioeconomic status in a Norwegian sample. Front. Public Health.

[21] Musumeci M.D., Cunningham C.M., White T.L. (2022). Disgustingly perfect: An examination of disgust, perfectionism, and gender. Motiv. Emot..

[22] Ge S., Chen C., Hewitt P.L., Flett G.L. (2023). Father-daughter and mother-son relationships: Parental bonding behaviours and socially prescribed perfectionism in young adults. Pers. Individ. Differ..

[23] Melero S., Morales A., Espada J.P., Fernández-Martínez I., Orgilés M. (2020). How does perfectionism influence the development of psychological strengths and difficulties in children?. Int. J. Environ. Res. Public Health.

[24] Hewitt P.L., Flett G.L. (1991). Perfectionism in the self and social contexts: Conceptualization, assessment, and association with psychopathology. J. Pers. Soc. Psychol..

[25] Tyler J.M., Panichelli-Mindel S.M., Sperrazza C., Levitt M.F. (2019). A pilot study exploring the relationship between perfectionism and anxiety in an urban middle school. J. Psychoeduc. Assess..

[26] Ferber K.A., Chen J., Tan N., Sahib A., Hannaford T., Zhang B. (2024). Perfectionism and social anxiety: A systematic review and meta-analysis. Clin. Psychol. Sci. Pract..

[27] Flett G.L., Coulter L.-M., Hewitt P.L., Nepon T. (2011). Perfectionism, rumination, worry, and depressive symptoms in early adolescents. Can. J. Sch. Psychol..

[28] Lin R.-M., Xie S.-S., Yan Y.-W., Chen Y.-H., Yan W.-J. (2017). Perfectionism and adolescent sleep quality: The mediating role of repetitive negative thinking. J. Health Psychol..

[29] Ying J., You J., Liu S., Wu R. (2021). The relations between childhood experience of negative parenting practices and nonsuicidal self-injury in Chinese adolescents: The mediating roles of maladaptive perfectionism and rumination. Child Abus. Negl..

[30] De Rosa L., Miracco M.C., Galarregui M.S., Keegan E.G. (2023). Perfectionism and rumination in depression. Curr. Psychol..

[31] Vicent M., Sanmartín R., Cargua-García N.I., García-Fernández J.M. (2022). Perfectionism and emotional intelligence: A person-centered approach. Int. J. Clin. Pract..

[32] Smith M.M., Sherry S.B., Ray C., Hewitt P.L., Flett G.L. (2021). Is perfectionism a vulnerability factor for depressive symptoms, a complication of depressive symptoms, or both? A meta-analytic test of 67 longitudinal studies. Clin. Psychol. Rev..

[33] Tran L., Rimes K.A. (2017). Unhealthy perfectionism, negative beliefs about emotions, emotional suppression, and depression in students: A mediational analysis. Pers. Individ. Differ..

[34] Liu L., Han Y., Lu Z., Cao C., Wang W. (2023). The relationship between perfectionism and depressive symptoms among Chinese college students: The mediating roles of self-compassion and impostor syndrome. Curr. Psychol..

[35] Peterson A., Smith-Morris C. (2024). Teen Perspectives on Suicides and Deaths in an Affluent Community: Perfectionism, Protection, and Exclusion. Int. J. Environ. Res. Public Health.

[36] Blackburn M., Molnar D.S., Zinga D. (2024). Simply the best? Bridging perfectionism in psychology and girlhood studies. J. Youth Stud..

[37] Stornæs A.V. (2023). Too Perfect to Be Healthy?: A Quantitative and Qualitative Study of Perfectionism, Expectations, and Mental Health Among Students Aged 13–16 in Specialized Sports, Performing Arts and Regular Lower Secondary School. Ph.D. Thesis.

[38] Tamannaeifar M., Hadadi S. (2023). The Mediating Role of Maladaptive Cognitive Emotion, Regulation Strategies In The Relationship Between Negative Perfectionism And Social Anxiety In Adolescent Girls. Int. J. Med. Investig..

[39] Díez M., Jiménez-Iglesias A., Paniagua C., García-Moya I. (2024). The Role of Perfectionism and Parental Expectations in the School Stress and Health Complaints of Secondary School Students. Youth Soc..

[40] Karababa A. (2020). The moderating role of hope in the relationship between maladaptive perfectionism and anxiety among early adolescents. J. Genet. Psychol..

[41] Kwarcinska K., Sanna K., Kamza A., Piotrowski K. (2022). Perfekcjonizm wteorii i badaniach. Prz. Psychol..

[42] Hadadi S., Tamannaeifar M. (2022). The comparison of maladjusted perfectionism, maladaptive cognitive emotion regulation, and rumination in adolescents with high and low social anxiety. Soc. Psychol. Res..

[43] Levine S.L., Green-Demers I., Werner K.M., Milyavskaya M. (2019). Perfectionism in adolescents: Self-critical perfectionism as a predictor of depressive symptoms across the school year. J. Soc. Clin. Psychol..

[44] Abdollahi A., Hosseinian S., Panahipour H., Allen K.A. (2021). Cognitive behavioural therapy as an effective treatment for social anxiety, perfectionism, and rumination. Curr. Psychol..

[45] Galloway R., Watson H., Greene D., Shafran R., Egan S.J. (2022). The efficacy of randomised controlled trials of cognitive behaviour therapy for perfectionism: A systematic review and meta-analysis. Cogn. Behav. Ther..

[46] Egan S.J., Wade T.D., Fitzallen G., O’Brien A., Shafran R. (2021). A meta-synthesis of qualitative studies of the link between anxiety, depression and perfectionism: Implications for treatment. Behav. Cogn. Psychother..

[47] Shafran R., Cooper Z., Fairburn C.G. (2002). Clinical perfectionism: A cognitive–behavioural analysis. Behav. Res. Ther..

[48] Rozental A. (2020). Beyond perfect? A case illustration of working with perfectionism using cognitive behavior therapy. J. Clin. Psychol..

[49] Egan S.J., Wade T.D., Shafran R., Antony M.M. (2016). Cognitive-Behavioral Treatment of Perfectionism.

[50] Steele A.L., Waite S., Egan S.J., Finnigan J., Handley A., Wade T.D. (2012). Psycho-education and group cognitive-behavioural therapy for clinical perfectionism: A case-series evaluation. Behav. Cogn. Psychother..

[51] Ward H. (2022). Effects of a single-session intervention targeting perfectionism in college students. Grad. Stud. J. Psychol..

[52] Bento C., Pereira A.I., Roque C., Saraiva J.G., Santos A. (2017). Longitudinal effects of an intervention on perfectionism in adolescents. Psicothema.

[53] Handley A.K., Egan S.J., Kane R.T., Rees C.S. (2015). A randomised controlled trial of group cognitive behavioural therapy for perfectionism. Behav. Res. Ther..

[54] Arana F.G., Miracco M.C., Galarregui M.S., Keegan E.G. (2017). A brief cognitive behavioural intervention for maladaptive perfectionism in students: A pilot study. Behav. Cogn. Psychother..

[55] Fairweather-Schmidt A.K., Wade T.D. (2015). Piloting a perfectionism intervention for pre-adolescent children. Behav. Res. Ther..

[56] Vekas E.J., Wade T.D. (2017). The impact of a universal intervention targeting perfectionism in children: An exploratory controlled trial. Br. J. Clin. Psychol..

[57] Szczucka K. (2010). Polski kwestionariusz perfekcjonizmu adaptacyjnego idezadaptacyjnego. Psychol. Spoleczna.

[58] Makara-Studzińska M., Tyburski E., Załuski M., Adamczyk K., Mesterhazy J., Mesterhazy A. (2022). Confirmatory factor analysis of three versions of the depression anxiety stress scale (DASS-42, DASS-21, and DASS-12) in Polish adults. Front. Psychiatry.

[59] Dragan M. (2016). Problemowe Picie Alkoholu Przez Mlode Kobiety: Rola Niekorzystnychdoswiadczen i Samoregulacji Emocji.

[60] George D., Mallery P. (2019). IBM SPSS Statistics 26 Step by Step: A Simple Guide and Reference.

[61] Malivoire B.L., Kuo J.R., Antony M.M. (2019). An examination of emotion dysregulation in maladaptive perfectionism. Clin. Psychol. Rev..

[62] Wright A., Fisher P.L., Baker N., O’Rourke L., Cherry M.G. (2021). Perfectionism, depression and anxiety in chronic fatigue syndrome: A systematic review. J. Psychosom. Res..

[63] Patterson H., Firebaugh C.M., Zolnikov T.R., Wardlow R., Morgan S.M., Gordon B. (2021). A systematic review on the psychological effects of perfectionism and accompanying treatment. Psychology.

[64] Lamarre C., Marcotte D. (2021). Anxiety and dimensions of perfectionism in first year college students: The mediating role of mindfulness. Eur. Rev. Appl. Psychol..

[65] Shafique N., Gul S., Raseed S. (2017). Perfectionism and perceived stress: The role of fear of negative evaluation. Int. J. Ment. Health.

[66] Finley A. (2020). The Relationship Among Perfectionism, Perceived Stress, and Coping in Baccalaureate Nursing Students. Ph.D. Dissertation.

[67] Vois D., Damian L.E. (2020). Perfectionism and emotion regulation in adolescents: A two-wave longitudinal study. Pers. Individ. Differ..

[68] Young K., Sandman C., Craske M. (2019). Positive and negative emotion regulation in adolescence: Links to anxiety and depression. Brain Sci..

[69] Dryman M.T., Heimberg R.G. (2018). Emotion regulation in social anxiety and depression: A systematic review of expressive suppression and cognitive reappraisal. Clin. Psychol. Rev..

[70] Schneider R.L., Arch J.J., Landy L.N., Hankin B.L. (2018). The longitudinal effect of emotion regulation strategies on anxiety levels in children and adolescents. J. Clin. Child Adolesc. Psychol..

[71] Park H., Jeong D.Y. (2015). Psychological well-being, life satisfaction, and self-esteem among adaptive perfectionists, maladaptive perfectionists, and nonperfectionists. Pers. Individ. Differ..

[72] Lipińska-Grobelny A., Bednarek K. (2021). Self-esteem and perfectionism versus procrastination. Kwart. Nauk. Fides Ratio.

[73] Smith M.M., Saklofske D.H., Yan G., Sherry S.B. (2017). Does perfectionism predict depression, anxiety, stress, and life satisfaction after controlling for neuroticism?. J. Individ. Differ..

[74] Hewitt P.L., Smith M.M., Ge S.Y.J., Mössler M., Flett G.L. (2022). Perfectionism and its role in depressive disorders. Can. J. Behav. Sci./Rev. Can. Sci. Comport..

